# Optogenetic activation of brainstem serotonergic neurons induces persistent pain sensitization

**DOI:** 10.1186/1744-8069-10-70

**Published:** 2014-11-19

**Authors:** You-Qing Cai, Wei Wang, Yuan-Yuan Hou, Zhizhong Z Pan

**Affiliations:** Department of Anesthesiology and Pain Medicine, The University of Texas MD Anderson Cancer Center, 1515 Holcombe Boulevard, Houston, Texas 77030 USA

**Keywords:** Serotonin, 5-HT, Optogenetic, Descending facilitation, Rostral ventromedial medulla, Pain

## Abstract

**Background:**

The rostral ventromedial medulla (RVM) is a key brainstem structure that conveys powerful descending influence of the central pain-modulating system on spinal pain transmission and processing. Serotonergic (5-HT) neurons are a major component in the heterogeneous populations of RVM neurons and in the descending pathways from RVM. However, the descending influence of RVM 5-HT neurons on pain behaviors remains unclear.

**Results:**

In this study using optogenetic stimulation in tryptophan hydroxylase 2 (TPH2)- Channelrhodopsin 2 (ChR2) transgenic mice, we determined the behavioral effects of selective activation of RVM 5-HT neurons on mechanical and thermal pain behaviors *in vivo*. We found that ChR2-EYFP-positive neurons strongly co-localized with TPH2-positive (5-HT) neurons in RVM. Optogenetic stimulation significantly increased c-fos expression in 5-HT cells in the RVM of TPH2-ChR2 mice, but not in wild type mice. Behaviorally, the optogenetic stimulation decreased both mechanical and thermal pain threshold in an intensity-dependent manner, with repeated stimulation producing sensitized pain behavior for up to two weeks.

**Conclusions:**

These results suggest that selective activation of RVM 5-HT neurons exerts a predominant effect of pain facilitation under control conditions.

## Background

The rostral ventromedial medulla (RVM), consisting of the nucleus raphe magnus (NRM), nucleus reticularis gigantocellularis and gigantocellularis pars alpha, is a key brainstem structure that relays processed information of pain from higher brain sites and exerts powerful descending modulation of spinal pain transmission through direct projections to spinal dorsal horn [1–3]. This descending pain modulation is functionally bi-directional, producing either pain inhibition or pain facilitation depending on behavioral conditions [4–8]. The RVM-mediated descending pain modulation plays a critical role in control of pain status for baseline pain responses under control conditions, pain inhibition induced by analgesic drugs, and pain sensitization in chronic pain conditions [2, 5, 6, 8].

Despite decades of research, the neurotransmitter systems of RVM that mediate the descending pain inhibition and pain facilitation remain unclear at present. RVM is a heterogeneous brainstem region containing diverse groups of neurons and neurotransmitter systems, such as glutamate, GABA, serotonin (5-HT), opioids and neuropeptides, including those neurons that project to the spinal cord [2, 3]. While RVM-descending pain inhibition has been the research topic since its discovery decades ago, studies in recent years are increasingly focusing on RVM-descending pain facilitation due to its now well recognized role in maintenance of a sensitized pain state in various conditions of chronic pain [2, 5, 6, 8].

5-HT cells, mainly in NRM, are one of the main cell groups in RVM and their spinal projections constitute a significant portion of descending pathways for pain modulation [3]. However, roles of RVM 5-HT cells in descending pain modulation are quite controversial with inconsistent results reported. Using the non-selective antagonist methysergide to block 5-HT receptors in the spinal dorsal horn where the RVM descending pathway projects, earlier studies indicate both pain-inhibitory and pain-facilitatory influence by the RVM 5-HT system [9–11]. Other studies with pharmacological or genetic methods to delete RVM 5-HT cells suggest that RVM 5-HT system has an inhibitory effect on pain [12, 13]. By down-regulating 5-HT synthesis and depleting 5-HT in RVM, recent studies show a major pain-facilitating role of the RVM 5-HT system under persistent pain conditions [14, 15]. These data support the notion that the RVM-descending 5-HT system may have both pain-inhibiting and pain-facilitating effects depending on behavioral state.

Recently, optogenetics has been increasingly used in neuroscience research to selectively and precisely control activity of a defined group of central neurons for determining their roles in behavioral functions in animals [16–20]. It also has been used in recent studies of pain [21–23]. In this study, we used the recently established TPH2-ChR2-EYFP transgenic mice, which express the light-sensitive protein channelrhodopsin-2 (ChR2) in central neurons containing tryptophan hydroxylase 2 (TPH2), the rate-limiting enzyme in 5-HT synthesis, as serotonergic cells [24]. Local optical stimulation was applied in the RVM of the transgenic mice to specifically activate RVM 5-HT neurons. In this experimental setting, we determined the behavioral effects of selective activation of RVM 5-HT neurons on thermal and mechanical pain responses in the freely moving transgenic mice *in vivo*.

## Results

### Selective expression of ChR2-EYFP in RVM 5-HT neurons in TPH2-ChR2 transgenic mice

**Figure 1:**
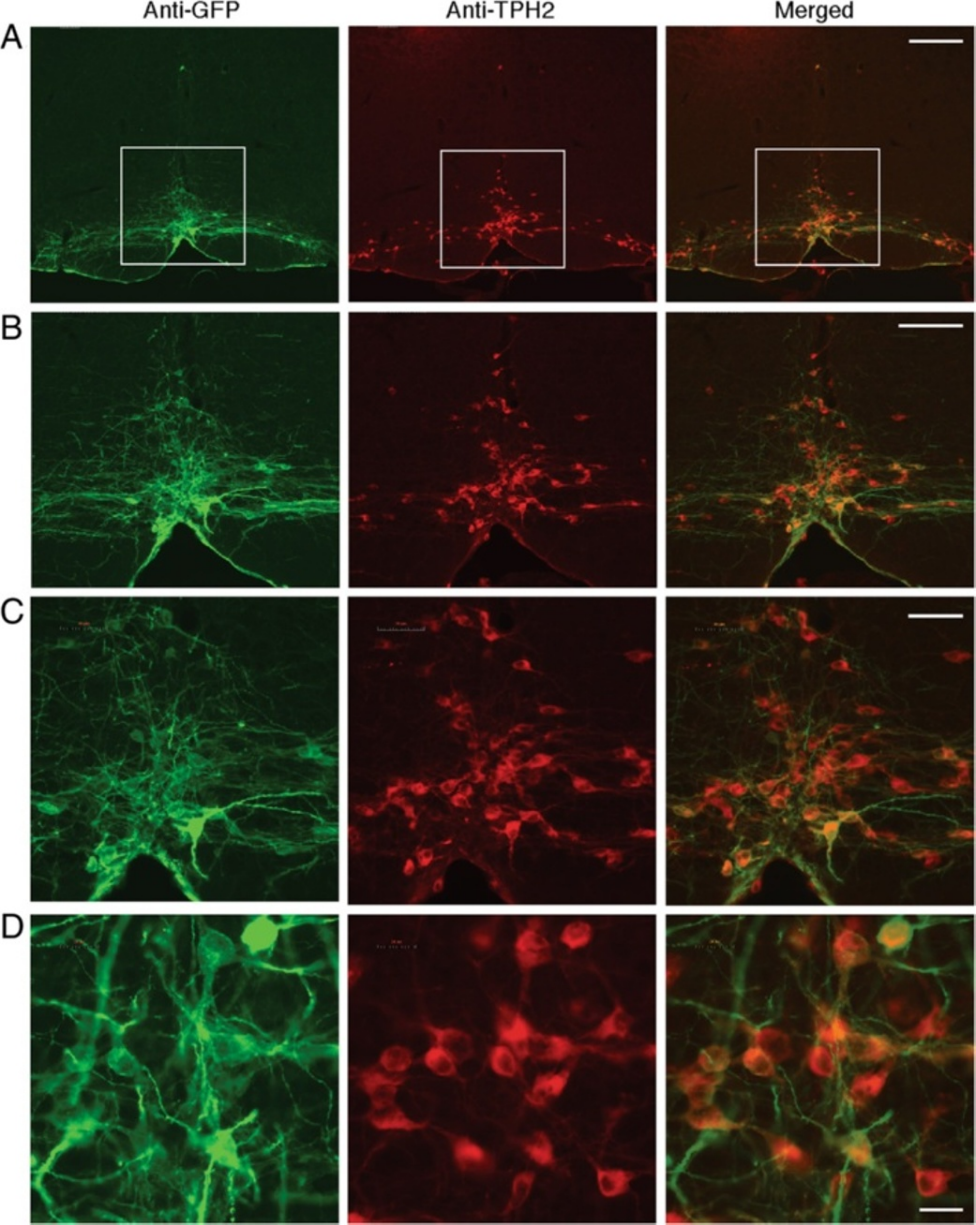
**Selective expression of ChR2-EYFP in serotoninergic (5-HT) neurons in the rostral ventromedial medulla (RVM) of TPH2-ChR2 transgenic mice.** Representative RVM images in increasing scales with anti-GFP staining for ChR2-EYFP-positive cells (green), anti-TPH2 staining for 5-HT cells (red), and merged images (yellow) from TPH2-ChR2 transgenic mice. The white squares encircle the magnified area in RVM shown in following images. Scale bars =200 μM **(A)**, 100 μM **(B)**, 50 μM **(C)** and 20 μM **(D)**.

We first examined the distribution of ChR2-EYFP-expressing cells in RVM, using anti-GFP antibodies and immunohistochemistry for EYFP staining as described in the original study of the transgenic mice [24]. We used THP2 as the marker of 5-HT cells in the CNS [15, 24] and examined their co-localization with ChR2-EYFP-expressing cells in RVM. As shown in Figure 1, TPH2-positive 5-HT cells were mainly localized in the nucleus raphe magnus (NRM) and lateral paragigantocellular nucleus, consistent with previous reports [25, 26]. These 5-HT neurons strongly co-localized with anti-GFP-labeled, ChR2-EYFP-expressing cells in RVM (267 GFP-positive cells out of 323 TPH2-positive neurons counted, 82.7%, from 6 slices of 3 different mice). Importantly, Nearly all GFP-positive neurons were also TPH2-positive, suggesting that ChR2-EYFP was specifically expressed in 5-HT neurons with no ectopic expression. This is consistent with the original report of TPH2-ChR2-EYFP transgenic mice with ChR2-EYFP exclusively expressed in 5-HT neurons in other brainstem raphe nuclei [24].

### Optogenetic stimulation increases c-fos expression in RVM 5-HT neurons

**Figure 2:**
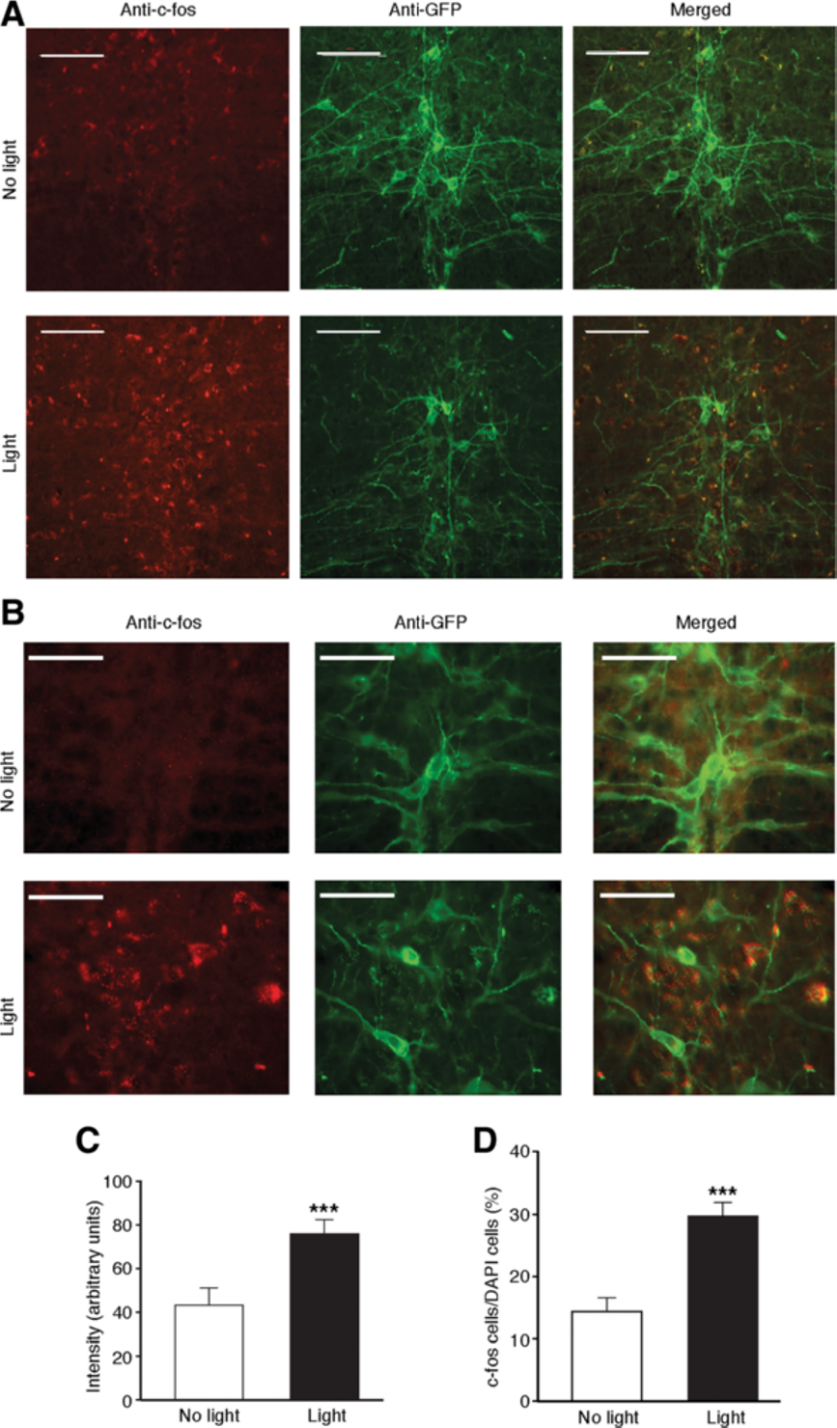
**Optical stimulation increases c-fos expression in RVM 5-HT neurons. (A)** Representative images showing anti-c-fos, anti-GFP and merged staining in the RVM of TPH2-ChR2 mice without (top row) and after single light stimulation (bottom row). Scale bars =100 μM. **(B)** Images in groups similar to those in **(A)** but in higher magnification (Scale bars =50 μM). **(C and D)** Summarized data of intensity of c-fos staining **(C)** and percentage of c-fos-positive cells vs. DAPI-positive cells **(D)** in the RVM of TPH2-ChR2 mice (9 slices from 3 different mice for each group). *** *p* <0.001 (unpaired t test).

We implanted an optical cannula in the RVM of TPH2-ChR2-Tg mice to activate ChR2-expressing 5-HT neurons with a single light stimulation (473 nm, 5 mW, 20 Hz, 15 ms) for 5 min, a stimulation protocol that has been shown to be selectively effective in these transgenic mice [24]. As reported in previous studies [17, 21], we examined changes in expression of the immediate early gene c-fos as the readout of neuronal activity. Using immunohistochemical microscopy, we quantified the light-induced changes in both general signal intensity of c-fos staining and the percentage of c-fos-positive cells in DAPI-positive cell population in the RVM of TPH2-ChR2-Tg mice. We found that, at 2 h after light stimulation, overall intensity of c-fos staining was significantly increased (t =4.581, *p* <0.001), and so was the number of c-fos-expressing cells when compared to RVM cells without the light stimulation (No light: 378 c-fos-positive cells vs. 2688 DAPI-positive cells; light: 1050 c-fos-positive cells vs. 3524 DAPI-positive cells; t =4.796, *p* <0.001, Figure 2). It is noteworthy that the c-fos expression was mainly increased in ChR2-expressing neurons, but it was also increased in some ChR2-negative neurons in RVM, indicating that other non-5-HT cells were possibly activated indirectly through optogenetic activation of 5-HT neurons in RVM.

**Figure 3:**
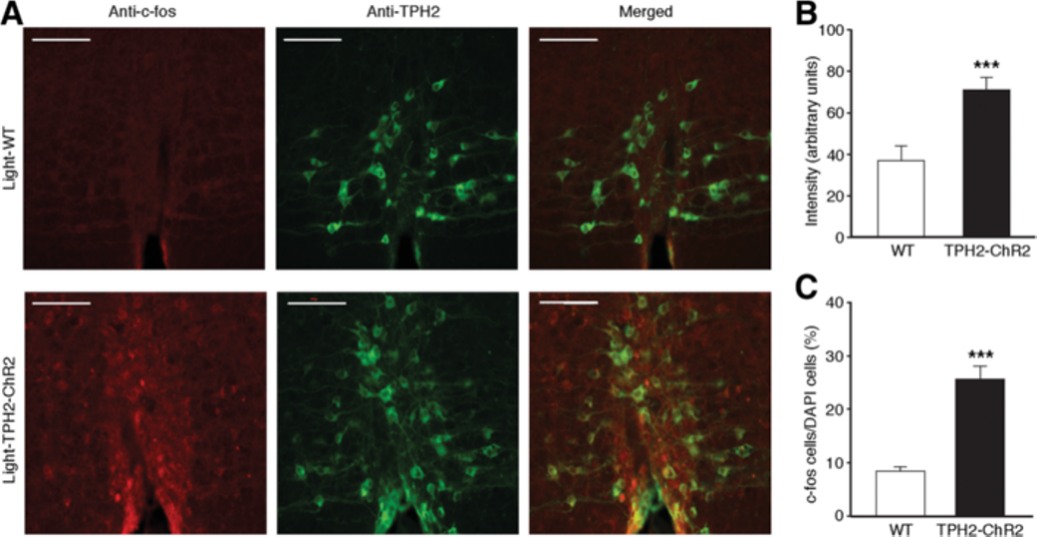
**Optical stimulation increases c-fos expression of RVM 5-HT neurons selectively in TPH2-ChR2 mice. (A)** Representative images showing anti-c-fos, anti-TPH2 and merged staining after single light stimulation in the RVM of WT mice (top row) and TPH2-ChR2 mice (bottom row). Scale bars =100 μM. **(B and C)** Summarized data of intensity of c-fos staining **(B)** and percentage of c-fos-positive cells vs. DAPI-positive cells **(C)** in the stimulated RVM of WT and TPH2-ChR2 mice (9 slices from 3 different mice for each group). *** *p* <0.001 (unpaired t test).

To verify that the increased activity of 5-HT neurons as measured by c-fos expression was indeed due to light activation of ChR2, but not to other non-specific stimulation from the light, we compared and analyzed similar data of c-fos-expression after the same light simulation in wild type (WT) mice and in TPH2-ChR2-Tg mice. Consistent with the above results, the optical stimulation significantly increased the intensity of c-fos expression (t =5.161, *p* <0.001) and number of c-fos-positive cells in the RVM of TPH2-ChR2-Tg mice when compared to those of WT mice (WT: 241 c-fos-positive cells vs. 2864 DAPI-positive cells; TPH2-ChR2: 770 c-fos-positive cells vs. 2946 DAPI-positive cells; t =6.798, *p* <0.001, Figure 3). Thus, it is likely that the optical stimulation selectively increased the activity of 5-HT neurons with possible secondary, downstream activation of some non-5-HT neurons in the RVM of the transgenic mice.

### Optogenetic stimulation of RVM 5-HT neurons induces persistent pain sensitization

**Figure 4:**
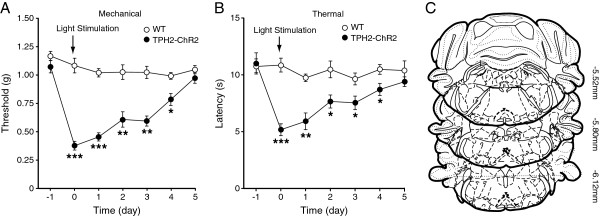
**Single optical stimulation in the RVM of TPH2-ChR2 mice induces persistent behaviors of pain sensitization. (A and B)** Changes in sensitivity of mechanical pain **(A)** and thermal pain **(B)** before (baseline) and after single light stimulation (arrow) given for 5 min on day 0 in WT and TPH2-ChR2 mice (n =5 each group). Pain tests were performed once daily. **(C)** Schematic drawing of brain slices illustrating tip positions of the optical cannulas (circle, WT; triangle, TPH2-ChR2). Numbers on the right are distance from Bregma. * *p* <0.05, ** *p* <0.01, *** *p* <0.001 (two-way ANOVA and Bonferroni’s *post hoc* analysis).

**Figure 5:**
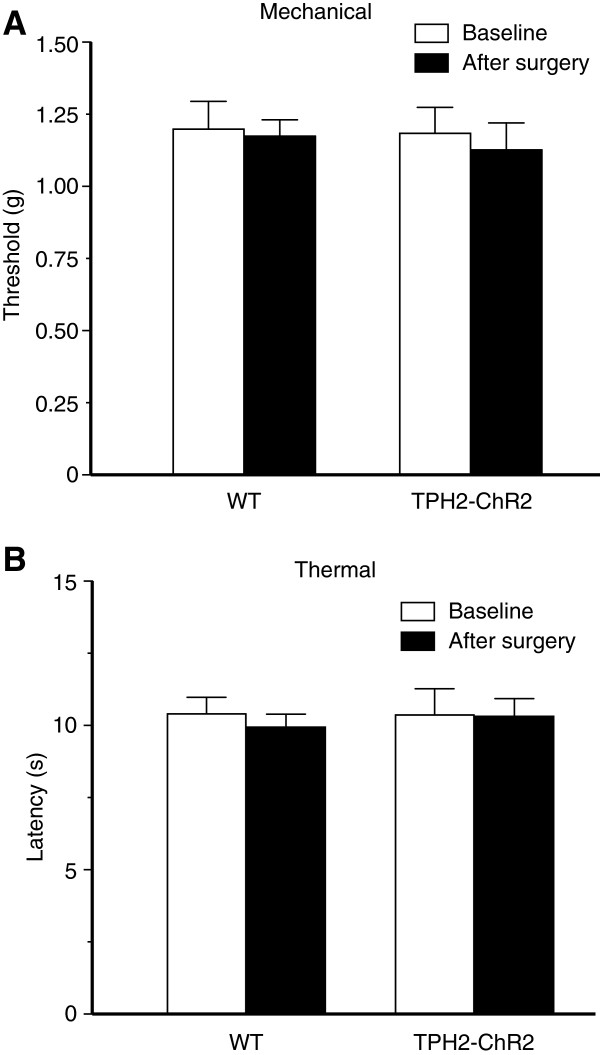
**Cannula implantation does not alter basal pain thresholds. (A and B)** Basal mechanical **(A)** and thermal **(B)** thresholds before (baseline) and 7 d after surgery for implantation of the optic fiber cannula in WT mice (n =5) and in TPH2-ChR2 mice (n =5) (paired and unpaired t test).

We then examined the behavioral effects of this optogenetic activation of RVM 5-HT neurons on pain behaviors in mice *in vivo*. We found that single optical stimulation of the same parameter induced persistent pain sensitization, with sustained decrease in pain thresholds measured by both the von Frey test for mechanical allodynia and by the paw-withdrawal test for thermal hyperalgesia (Figure 4A: F_(6,48)_ =15.88, *p* <0.001; Figure 4B: F_(6,24)_ =5.417, *p* =0.0012). The pain sensitization lasted about 4 days in both mechanical allodynia and thermal hyperalgesia. In contrast, the same optical stimulation failed to induce significant changes in the basal pain thresholds of both mechanical and thermal tests in control WT mice. Figure 4C depicts tip positions of the optical cannulas in brainstem slices for light stimulation in these behavioral pain tests. In addition, implantation of the optic fiber cannula did not alter the basal mechanical or thermal thresholds, excluding a potential effect of the surgery itself (Figure 5). These behavioral results suggest that activation of RVM 5-HT neurons induces a persistent pain-facilitating effect.

**Figure 6:**
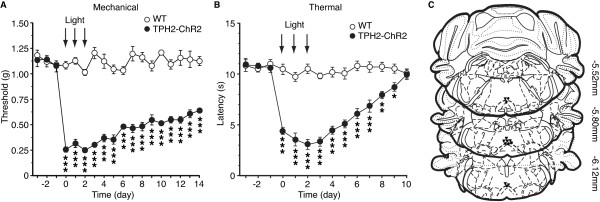
**Repeated optical stimulation in the RVM of TPH2-ChR2 mice induces long-lasting behaviors of pain sensitization. (A and B)** Changes in sensitivity of mechanical pain **(A)** and thermal pain **(B)** before (baseline) and after repeated light stimulation (arrows) given for 5 min once daily for 3 days on day 0 through day 2 in WT (n =6) and TPH2-ChR2 mice (n =7). Pain tests were performed once daily. **(C)** Schematic drawing of brain slices illustrating tip positions of the optical cannulas (circle, WT; triangle, TPH2-ChR2). Numbers on the right are distance from Bregma. * *p* <0.05, ** *p* <0.01, *** *p* <0.001 (two-way ANOVA and Bonferroni’s *post hoc* analysis).

To determine whether the pain sensitization was dependent on the intensity of optical stimulation, we reduced the light intensity to 2.5 mW with other stimulation parameters unchanged (473 nm, 20 Hz, 15 ms for 5 min) and repeated the stimulation protocol once daily for three consecutive days. Consistent with the result of single light stimulation, we found that this repeated optical stimulation produced stronger pain sensitization in both mechanical allodynia and thermal hyperalgesia that lasted much longer than that after single stimulation, with significant mechanical allodynia remaining after 14 days, the latest time point of measurement (Figure 6A: F_(17,187)_ =21.06, *p* <0.001; Figure 6B: F_(13,143)_ =20.73, *p* <0.001). These results further support the notion that activation of RVM 5-HT neurons induces long-lasting pain sensitization.

## Discussion

In this study, we have shown that selective optical activation of RVM TPH2-expressing 5-HT neurons produces persistent behaviors of pain sensitization as mechanical allodynia and thermal hyperalgesia. The finding that this pain sensitization lasts for days to weeks suggests that the 5-HT neurons in RVM are able to exert powerful and long-lasting facilitatory influence on spinal processing of pain, leading to a persistent state of pain sensitization.

Since RVM 5-HT neurons constitute one of the major neurotransmission systems in the brainstem descending pain-modulating pathways [3], understanding of their functions is important to reveal neural mechanisms for central modulation of pain under normal and pathological conditions. In this regard, almost all previous studies used an approach of loss-of-function by pharmacologically or genetically inhibiting RVM 5-HT cells. This approach requires creation of a pre-existing condition, either stimulus/drug-induced antinociception or inflammation/injury-induced pain sensitization. As such, RVM 5-HT cells were shown to be antinociceptive, as their inhibition reduced the antinociception, or to be pronociceptive, as their inhibition attenuated the pain sensitization [9–11, 14, 15, 27, 28]. Interestingly, while earlier studies tend to focus on the antinociceptive role of RVM 5-HT cells, recent studies with more specific targeting of 5-HT cells and 5-HT receptor subtypes report a pronociceptive effect of RVM 5-HT cells under persistent pain conditions [14, 15]. The current study used a unique approach of acute functional activation to selectively increase activity of RVM 5-HT neurons under normal conditions and reveals a pronociceptive effect on normal pain responses without pre-existing conditions and consequently induced adaptive changes within the RVM systems. This effect may suggest that the group of RVM 5-HT neurons, if activated as a whole, has a net or predominant pain-facilitating effect under various behavioral states. Some early studies used RVM administration of 5-HT and observed either an antinociceptive effect or no effect on pain threshold [29–31]; however, 5-HT may activate or inhibit RVM cells, depending on the 5-HT receptor subtype the cells contain, and the cell population affected by 5-HT administration is likely different from that of this study, which contains 5-HT and likely releases 5-HT onto target cells within RVM and in their projection area of spinal dorsal horn.

The cellular mechanism by which this selective activation of RVM 5-HT neurons induces pain sensitization is still unclear. Within RVM, although there is discrepancy regarding RVM cell classes that contain 5-HT, both anatomical and electrophysiological studies demonstrate that a significant portion of spinally projecting RVM 5-HT cells contain mu-opioid receptor (MOR) [32, 33]. Since MOR-containing RVM cells have been well recognized as a cell class that exerts pain facilitation [2], optical activation of this group of 5-HT cells would account for the sensitized pain behaviors we observed. It is noteworthy that this optical activation of 5-HT cells likely also indirectly activated other non-5-HT cells, including those with spinal projections, through synaptic connections in RVM, as shown by increased c-fos expression in some non-ChR2-expressing RVM cells. This is consistent with the previous report of feed-forward connections between 5-HT cells and non-5-HT cells in RVM-descending control pathways [25]. Nevertheless, activation of 5-HT neurons and descending pain facilitation appears predominant over potential descending influence from other non-5-HT cells after the optical stimulation in RVM. It is likely that this pain sensitization involves 5-HT that is released from the optically activated, spinally projecting RVM 5-HT cells and acts on 5-HT receptor-expressing neurons in spinal dorsal horn. Numerous studies using pharmacological antagonists or genetic disruption of specific 5-HT receptor subtypes have shown that spinal 5-HT1 and 5-HT2 receptors are predominantly antinociceptive whereas spinal 5-HT3 receptors have been shown to mediate mostly pain sensitization with some reports of pain inhibition in animal models [3, 34–41].

The TPH2-ChR2 mice provide a unique tool to control activity of 5-HT neurons in the brain *in vivo* with temporal and spatial precision. In these mice, the blue light has been shown to evoke reliable firing of action potentials at up to 20 Hz in TPH2-ChR2-expressing neurons [24]. In a recent study of pain-induced sleep disorder, similar optical stimulation was used to activate defined 5-HT neurons in the dorsal raphe nucleus (DRN) of TPH2-ChR2 mice *in vivo* [23]. In the current study, ChR2-EYFP-positive RVM neurons were identified as 5-HT cells, as they strongly overlapped with TPH2-containing neurons, consistent with a recent report showing that TPH2 completely co-localizes with 5-HT in RVM cells [15]. Our optical stimulation significantly increased c-fos expression when compared to that without the stimulation in the RVM cells of TPH2-ChR2 mice and the same stimulation failed to alter c-fos expression in the RVM cells of WT mice, indicating that the optical stimulation likely activates RVM 5-HT cells, leading to an RVM 5-HT cell-initiated descending effect of pain facilitation. Of note is that ChR2-EYFP expression is also present in DRN 5HT neurons that may project to RVM in the TPH2-ChR2-Tg mice. Thus, it cannot be ruled out that the optical stimulation in RVM also activated ChR2-expressing never fibers from DRN.

This RVM 5-HT system of pain facilitation may play an important role in the mechanisms for the development of chronic pain. Many recent studies using animal models of chronic pain have shown that selective inhibition of 5-HT functions in RVM or in RVM-projected spinal dorsal horn attenuates sensitized pain behavior [14, 15]. This suggests that RVM 5-HT function may be upregulated in chronic pain conditions. Indeed, RVM TPH2 protein level is increased in a rat model of neuropathic pain and persistent inflammatory pain increases spontaneous activity of some TPH-containing 5-HT cells in RVM [15, 42]. Whereas a similar protocol of optical stimulation (20 Hz, 10 ms, 9 mW) in the DRN of TPH2-ChR2-Tg mice evokes 5-HT release in its projection area of prefrontal cortex [23], it remains to be demonstrated what are the spinal 5-HT receptor subtypes involved in the pain sensitization if 5-HT is released from RVM-descending 5-HT pathways activated by the optical stimulation. It is also unclear how persistent pain conditions may activate the RVM 5-HT function and one possibility is that RVM 5-HT neurons are activated by noxious stimuli through a spinoreticular pathway [26]. As chronic pain is considered as the result of imbalanced shift in RVM modulation from pain inhibition to pain facilitation [43], the RVM 5-HT system may play an important role in this shift and could be a therapeutic target in the treatment of chronic pain.

## Conclusions

In this study, we show that optogenetic activation of tryptophan hydroxylase 2-expressing serotonergic neurons in rostral ventromedial medulla *in vivo* induces a long-lasting (days – weeks) pain sensitization measured as mechanical allodynia and thermal hyperalgesia. It suggests a predominant pain-facilitating role of the brainstem 5-HT neurons in spinal pain processing.

## Methods

### Animals

Male heterozygous TPH2-ChR2-EYFP BAC transgenic mice were purchased from the Jackson Laboratory. The male transgenic mice were crossed with female C57BL/6 J mice to obtain TPH2-ChR2-Tg mice and WT littermates. This strain was generated by Zhao et al. and ChR2-EYFP is selectively expressed in serotonergic neurons recognized by anti-GFP antibodies with no ectopic expression in brainstem [24]. Mice were housed in groups of five with food and water available *ad libitum*, and a 12 h light/dark cycle. Behavioral experiments and tests were performed between 8:00 a.m. and 18:00 p.m. All procedures involving the use of animals conformed to the guidelines set by the Institutional Animal Care and Use Committee of MD Anderson Cancer Center.

### Implantation of optical fiber cannula and optical stimulation

A mono fiber-optic cannula (Doric Lenses Inc., Canada) was stereotaxically implanted just above the RVM (AP, 5.8 mm; L, 0.0; V, 5.3 mm). Animals were single housed after the implantation surgery and allowed to recover for 7 days before behavioral tests. For optical stimulation, the implanted cannula was connected to a 473 nm DPSS laser (Shanghai Laser & Optic Century Co., China) through a fiber-optic patch cord with a rotary joint for free movement of the animal. Blue light pulses of 20 Hz, 15 ms, 5 mW with 17.68 mW/mm^2^ or 2.5 mW with 8.84 mW/mm^2^ were delivered to the RVM for 5 min once or once daily for 3 d. Intensity of the fiber-optic light at the end of fiber was verified before and after each experiment by a power meter (PM-100D, Thor Labs). All laser outputs were controlled by a Master-8 pulse stimulator (A.M.P.I).

### Von Frey test for sensitivity of mechanical pain

Mice were extensively handled and habituated to the test environment and test apparatus for at least 3 d before all behavioral tests. A mouse was placed in a plastic box with mesh floor and allowed to acclimate for 1 h. A series of calibrated von Frey filaments were applied perpendicularly to the plantar surface of a hind paw with sufficient force to bend the filament for 6 s. Brisk hindpaw movements (withdrawal or flinching) were considered to be a positive response. The tactile stimulus producing a 50% likelihood of withdrawal was determined by the “up-down” calculating method [44, 45]. Basal threshold was measured as baseline before optical stimulation. A 473 nm blue light was delivered to the RVM for 5 min with the single or repeated stimulation protocol described above. The hindpaw withdrawal responses were measured twice with a 5-min interval 10 min after light stimulation.

### Analgesia test for sensitivity of thermal pain

A mouse was placed in a Plantar Test Instrument (Model 37370, Ugo Basile, Italy). Response to an infrared heat stimulus was measured with a Hargreaves apparatus and only quick hindpaw withdrawal (with or without licking) was counted as a response [46]. Latency to the paw withdrawal was recorded automatically by the Instrument. The latency before and after the optical stimulation was measured twice with a 5-min interval 50 min after light stimulation. The data represent the average value for withdrawal latencies of both right and left hindpaw measured alternatively.

### Immunohistochemistry

Mice were deeply anesthetized with pentobarbital and transcardially perfused with 20 ml heparinized saline and subsequently with 30 ml ice-cold 4% paraformaldehyde in 1 × PBS (pH 7.4). The brain and spinal cord were removed and post-fixed in 4% paraformaldehyde overnight at 4°C, followed by dehydration with 30% sucrose in 1 × PBS. Tissues were sectioned into 30-μm thick coronal sections with a cryostat at -20°C. Sections were blocked with 5% normal donkey serum in PBS containing 0.3% Triton X-100 and incubated overnight with primary antibodies (rabbit anti-GFP antibody, 1:1000 dilution, A11122, Invitrogen; mouse anti-TPH2 antibody, 1:500 dilution, T0678, Sigma; and rabbit anti-c-Fos antibody, 1:1000, sc-52, Santa Cruz). Sections were then rinsed and incubated with the Alexa Fluor-conjugated secondary antibodies (1:500, Alexa Fluor 488 and 568, Invitrogen), and were mounted on slides, dried and coverslipped with ProLong Gold antifade reagent for staining with the fluorescent reporter 4′,6-diamidino-2-phenylindole (DAPI, Invitrogen). The stained sections were examined with an Olympus BX51 fluorescence microscope. Intensity of fluorescence signals for c-fos staining in images was automatically quantified and analyzed by HCImage software (Hamamatsu Corporation, PA). For each group, 9 slices from 3 different mice were selected and in each slice, 4 randomly selected areas (250 μm × 250 μm) in the TPH2-positive or GFP-positive areas within RVM were chosen for cell counts. The number of c-fos-positive cells and DAPI-positive cells in the selected areas was counted manually by experimenters blind to experimental conditions.

### Statistical analysis

For data of pain tests, two-way ANOVA for repeated measures with *post hoc* analysis of the Bonferroni method was used to determine statistical significance in group treatment and between-group interactions at each time point. Unpaired Student’s *t* test was used for comparisons of c-fos data between groups. A *p* value of <0.05 was considered statistically significant. All statistical analyses were performed with the Prism software version 6.0 (GraphPad Software). Data are presented as mean ± S.E.M.

## Abbreviations

ChR2: Channelrhodopsin-2
DAPI: 4′,6-diamidino-2-phenylindole
DRN: Dorsal raphe nucleus
MOR: Mu-opioid receptor
NRM: Nucleus raphe magnus
RVM: Rostral ventromedial medulla
TPH2: Tryptophan hydroxylase 2
WT: Wild-type.
